# Scalable High-Resolution Single-Pixel Imaging via Pattern Reshaping

**DOI:** 10.3390/s24144689

**Published:** 2024-07-19

**Authors:** Alexandra Osicheva, Denis Sych

**Affiliations:** Terra Quantum AG, Kornhausstrasse 25, 9000 St. Gallen, Switzerland; densy@terraquantum.swiss

**Keywords:** single-pixel imaging, single-pixel camera, image reconstruction, Hadamard matrices, high-resolution imaging, structured detection

## Abstract

Single-pixel imaging (SPI) is an alternative method for obtaining images using a single photodetector, which has numerous advantages over the traditional matrix-based approach. However, most experimental SPI realizations provide relatively low resolution compared to matrix-based imaging systems. Here, we show a simple yet effective experimental method to scale up the resolution of SPI. Our imaging system utilizes patterns based on Hadamard matrices, which, when reshaped to a variable aspect ratio, allow us to improve resolution along one of the axes, while sweeping of patterns improves resolution along the second axis. This work paves the way towards novel imaging systems that retain the advantages of SPI and obtain resolution comparable to matrix-based systems.

## 1. Introduction

Single-pixel imaging (SPI) is a method for obtaining images without the need for matrix photodetectors. The key idea behind SPI is the use of a sequence of light patterns and a photodetector that measures correlations between the object of interest and the light patterns. The image can be reconstructed from the measured data and the knowledge of the spatial structure of employed light patterns.

The simplest way to implement SPI is raster scanning, similar to Nipkow disks in early television. In this case, an image is overlapped with a sequence of patterns, each of which is completely dark except for only one bright pixel that moves from pattern to pattern. A complete system of Nipkow patterns can be represented by an identity matrix, where each row is a one-dimensional folding of a corresponding two-dimensional light pattern. The image is sampled in a point-by-point fashion, and essentially no computation or data-processing is required: the brightness of each pixel is directly measured by the corresponding pattern.

One can significantly increase the signal-to-noise ratio by using a sequence of light patterns that forms a matrix with more than one non-zero elements in each row [1]. A very convenient system of patterns can be constructed from orthogonal matrices, in particular, Hadamard matrices. The initial proposal dates back to the late 1940s [2], followed by numerous improvements and experimental realizations in the early 1970s, giving rise to the area of Hadamard-transform optics and spectrometers [3,4,5,6,7].

A rediscovery of SPI in the mid-1990s happened in the domain of quantum optics, widely known nowadays as “ghost imaging” [8]. Instead of Hadamard-based patterns, the object is illuminated by a sequences of random patterns, generated via non-linear light scattering. Eventually, random light patterns can be also generated in a classical way, and the computation of images can be performed using a compressive sampling technique [9,10]. Compressive sampling provides a way to reduce the number of light patterns below the number of image pixels for precise image reconstruction, and stimulated the development of single-pixel cameras as practical SPI devices.

SPI has numerous advantages over the traditional matrix-based approach in imaging regimes where the matrix detector technology is hard to implement [11]. Such applications include imaging at non-visible wavelengths (e.g., infrared imaging [12,13], terahertz imaging [14]), single-photon imaging [15], and 3D-imaging [16], to name a few.

One of the challenges in SPI is obtaining high resolution. The typical resolution in SPI is relatively low (e.g., 32×32) and rarely exceeds 128×128. This limitation is due to several reasons. Higher resolution requires a higher number of light patterns, which increases the object exposure time. The minimum exposure time for one pattern is also lower-limited by a pattern refresh rate and a reasonable signal-to-noise ratio of the detector. Next, even allowing for a long exposure time, the practical limit for resolution comes from the computational resources, since the image is the result of data processing. For example, compressive sampling is a very attractive approach due to a lower number of required light patterns, but resolution scaling is limited due to the demanding computational algorithms [17]. The use of neural networks also allows image resolution to be increased under a fixed number of measurements, but real-world experiments provide rather moderate image resolution [18]. Eventually, one can exploit particular properties of pattern display hardware to extend the resolution [19].

A possible solution for high-resolution SPI is the use of fast computational algorithms together with properly chosen light patterns and sampling procedures. In this work, we demonstrate a simple experimental design for achieving scalable high-resolution SPI without complicated computations. Unlike the above-mentioned approaches to increase image resolution, we do not rely only on modifications of the image reconstruction algorithms, but rather introduce a complimentary procedure to ensure the scalability of the overall imaging system. Each light pattern in our setup consists of an “active” part, while the rest pixels outside the active part are “idle”. The active part has a configurable rectangular form and is filled by a reshaped row of a Hadamard matrix. The image is reconstructed in two steps. First, we run the complete sequence of light patterns within the active area. Second, we sweep the active area across the entire image by a rotating mirror. As a result, the number of different light patterns programmed in the spatial light modulator in this approach is rather small, while the resulting resolution can be rather high. In particular, we demonstrate the use of light patterns based on a 64×64 Hadamard matrix, which normally provides 8×8 image resolution, to obtain 64×112 image resolution, with a clear path for further scale-up.

## 2. Theoretical Background

The general idea of SPI with the use of a complete set of measurements can be described as follows [3]. We denote the total number of pixels of the image by *N* and enumerate them by a single one-dimensional index. Here, we do not specify the image aspect ratio and consider it as a one-dimensional object irrespective of its actual shape. Let *O* be a vector of length *N* containing the brightness value of each pixel of an object of interest, *S* be a vector of length *N* containing the measured signal values for a given sequence of light patterns, and *P* be a binary matrix of size N×N, each row of which corresponds to one light pattern that consists of 0 (“closed” pixel) and 1 (“open” pixel). Then the measurement of the signal *S* from the object *O* is described in matrix form as S=P·O.

The image of the object can be reconstructed as $O=P^{-1}\cdot S$, provided that the matrix *P* is non-degenerate. In fact, realistic data acquisition is always noisy, which distorts the obtained image: $\tilde{O}=P^{-1}\cdot \left(S+\Delta S\right)$, where $\tilde{O}$ is a noise-distorted image of the object, *S* is a signal in the ideal noiseless case, and ΔS is a vector of length *N* representing noise for each element of the signal *S*.

In our experiment, we use patterns based on a 64×64 Hadamard matrix *H* in a Sylvester construction [20]. This choice is due to the easy form of the inverse matrix $H\cdot H^{T}=NI_{N}$, where $H^{T}$ is the matrix transpose and $I_{N}$ is the identity matrix.

The Hadamard matrix *H* consists of 1 and −1, while the set of patterns *P* must be constructed from 0 and 1. We represent the Hadamard matrix *H* as $H=H^{+}-H^{-}$, where $H^{+}$ and $H^{-}$ consist only of 0 and 1. In order to make a measurement corresponding to the Hadamard matrix, we need to separately measure two signals, the first from its positive part $H^{+}$ and the second from its negative part $H^{-}$, and then subtract the second signal from the first one. Then, the image can be reconstructed as

(1) $$O=H^{-1}\cdot \left(S^{+}-S^{-}\right),$$

where $S^{+}$ and $S^{-}$ are signals from sequences of patterns corresponding to the matrices $H^{+}$ and $H^{-}$. Here, we see another argument to use Hadamard matrices instead of apparently much simpler raster scanning SPI [15], expressed by the identity matrix, namely, the larger signal-to-noise ratio due to the larger number of open pixels.

Besides the fact that the inverse matrix is known, Hadamard matrices have maximum determinant among all matrices with elements normalized to 1 (i.e., absolute values of all elements are less than or equal to 1), which makes such pattern system very robust to noise. As shown in [1,21], for any choice of matrix *P* that consists of such elements, the minimum possible variance of each pixel brightness value can be achieved if P=H.

## 3. Experimental Setup

**Figure 1:**
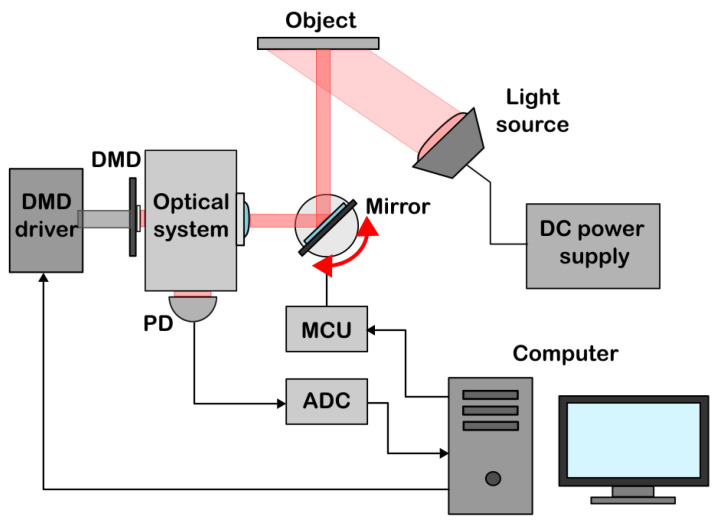
The scheme of the experimental setup. The object is illuminated using a halogen lamp connected to a DC power supply. The light from the object is reflected from a mirror which is mounted on a stepper motor and can be rotated in the horizontal plane. The stepper motor angle of rotation is adjusted using a microcontroller unit (MCU). The part of the light reflected from the mirror is focused by an optical system on an array of micromirrors in a digital micromirror device (DMD). The patterns on the DMD are switched using a DMD driver at a frequency of 9523 Hz. The part of the light corresponding to a pattern displayed on the DMD is measured by a single-pixel photodetector (PD). The signal from the photodetector is amplified and measured using an analog-to-digital converter (ADC). The MCU, ADC, and DMD driver are connected to a computer.

The experimental setup for obtaining images is shown in Figure 1. We use black-and-white images on a piece of paper as objects of interest. The object is illuminated by a halogen lamp connected to a DC power supply. The level of the illumination can be varied by changing the voltage of the power supply. A light diffuser is installed in front of the lamp to ensure uniform illumination. It is also possible to operate the setup in natural daylight conditions, provided its level is constant during the measurement.

The light from the object is reflected from the mirror. The mirror can be rotated in the horizontal plane in order to change the visualized area of the object during the measurement. The mirror is rotated using a stepper motor controlled by a microcontroller unit (MCU). The stepper motor completes a full rotation in 12,800 steps, which allows for the accurate adjustment of a position of the mirror.

The light reflected from the mirror is focused by a specially constructed optical system onto the array of micromirrors of a Texas Instruments DLP6500 digital micromirror device (DMD) that consists of 7.56 µm micromirrors arranged as a 1920×1080 array. This DMD operates as a spatial light modulator: the micromirrors can be fixed in either of two angular positions $12^{{}^{\circ}}$ or $-12^{{}^{\circ}}$ relative to the plane of the array of micromirrors, reflecting the incident light in one direction or another. An angular position is chosen in accordance with a given pattern: “open” and “closed” pixels correspond to $12^{{}^{\circ}}$ and $-12^{{}^{\circ}}$ mirror positions. The patterns are displayed on the DMD using the DLPC900 driver. The experiment uses the technically minimum possible display time of one pattern, equal to 105 µs, which corresponds to the maximum 9523 Hz switching frequency of binary patterns.

The part of the light corresponding to a displayed pattern is reflected from the DMD towards a single-pixel photodetector. The photodetector signal is amplified and measured using a 14-bit analog-to-digital converter (ADC). The measurement frequency of the ADC is 2 MHz, which corresponds to 210 points per pattern and allows for accurate acquisition of the signal value for the pattern by averaging the obtained values.

The MCU, ADC, and DMD driver are connected to a computer that controls the measurement process. A dedicated Texas Instruments PC software is used to load patterns into the DMD control unit. Output triggers of the DMD driver are used to synchronize the displayed sequence of patterns and measurements of the signal. The amplified signal from the photodetector is averaged for each pattern and used to reconstruct the image using Equation (1). The obtained image is displayed on the computer screen.

The DMD has some features that directly affect the experiment. The driver is capable of holding up to 400 binary patterns in its memory. Also, according to the DMD data sheet, it is advisable to demonstrate patterns such that each micromirror is in equal time in each of the two possible positions. To comply with these recommendations, the sequence of patterns demonstrated during the operation of the experimental setup contains negatives of the original patterns, which are used only to balance the micromirror timing. The presence of these negatives inevitably leads to further restrictions on the maximum number of useful patterns to 200.

## 4. Variable Resolution via Hadamard Pattern Reshaping

The image reconstruction process is quite simple: it consists of averaging the signal for each pattern, pairwise subtraction of the averaged signals, and multiplication by the inverse matrix, according to Equation (1). Using our setup, we can quickly reconstruct small-size images, for example, of size 8×8, in less than 0.1 s. Fast acquisition times enable us to obtain high-resolution images using multiple measurements of a sequence of lower-resolution patterns, as described below.

Hadamard-based patterns can be obtained for various image resolutions, provided the corresponding Hadamard matrix exists [22]. Moreover, we can form patterns for different aspect ratios of images, while the image reconstruction process remains the same. For example, using a 64×64 Hadamard matrix, we can form patterns of sizes 8×8, 16×4, 32×2, and 64×1.

In order to increase the image resolution, one can use larger Hadamard matrices and the larger number of patterns. In our work, we use non-squared patterns and the rotating mirror to obtain high-resolution images using a fixed limited number of patterns. The mirror allows us to use the same pattern sequence for step-by-step scanning of an object in a horizontal plane as follows.

The DMD displays a sequence of patterns which consist of active and idle parts. The active part of each pattern has a rectangular form: the height is equal to the height of the image and the width is less than the width of the image. The active part is filled by a reshaped row of the Hadamard matrix. The aspect ratio used for reshaping is chosen according to the vertical image resolution and active part size. The idle part is filled by closed pixels. Further in the text, “pattern size” implies the size of the active part.

**Figure 2:**
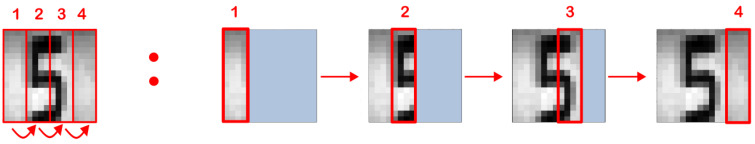
Schematics of discrete sweep image reconstruction. The process of obtaining an image of a handwritten digit «5» consists of 4 scanning steps in a horizontal plane with a sequence of 16×4 patterns. The part of the object that is visualized at the current step is shown by the bold red frame. The part of the image that is missing at the current step is filled in light blue.

The discrete sweep image reconstruction algorithm consists of several steps. First, the mirror rotation angle fixes a certain part of the object in the horizontal plane. Second, the pattern sequence measurement is carried out, and part of the image is reconstructed and saved. After that, the mirror is rotated to the next position, and the process is repeated. The final image is constructed from the saved parts and displayed on the computer screen. An example of the setup operation is shown in Figure 2: a sequence of 16×4 patterns is used to obtain a 16×16 image in 4 steps.

The final horizontal image resolution is equal to the product of the horizontal size of the patterns by the number of steps. The vertical resolution is fixed by the pattern sequence. In order to obtain a higher final resolution, we should make the vertical resolution as high as possible. Thus, this method can be used to obtain high-resolution images using a small number of patterns. The image acquisition time depends mainly on the number of measurement steps, and is limited by the operation of the electronics controlling the mirror and the timing of the corresponding interfaces.

To speed up the visualization process, we developed continuous sweep mode. The mirror continuously moves at a specially selected speed, and pattern sequence measurements (steps) are carried out at properly chosen fixed time intervals such that for each subsequent measurement, we visualize a new part of the object. This continuous sweep mode works well with patterns whose height significantly exceeds their width. Importantly, the image acquisition time in continuous sweep mode is significantly reduced compared to discrete sweep mode. For example, to obtain a 32×48 image in discrete sweep mode, we need 24 steps, which takes 40 s. In contrast, when using continuous sweep mode, we obtain a 64×112 image in 112 steps, which takes 11 s.

During the measurement process, it is important to maintain a constant level of illumination; otherwise, each separately measured part of the image has a different brightness, which worsens the quality of the resulting image and requires additional processing.

The maximum image resolution is bounded by the total number of patterns and the total data acquisition time. To estimate the upper limit on the final image resolution, we fix the data acquisition time to 1 s.

In our work, we use a complete system of equations, i.e., the image sampling rate is 1; hence, we need 64 Hadamard patterns (which are realized via 128 binary patterns) to reconstruct 64 pixels at a time. The additional 128 color-inverted patterns are technical requirements of the DMD. The pattern switching rate is around 9.5 kHz; thus, we reconstruct app. 37 frames per second and obtain an effective image resolution of 64×37. There are faster DMDs that deliver, e.g., a 35 kHz pattern switching rate; thus, the image resolution can be further increased to app. 64×136.

To achieve even higher image resolution with a fixed number of patterns, one can use more complicated image reconstruction methods such as compressive sampling or neural networks. The sampling rate can be reduced to a few percent while keeping reasonable image quality. For example, the use of a generative adversarial network with a 5% sampling rate in [18] would allow us to increase the final image resolution to app. 320×480 using our sweeping method. We note, however, that the use of either compressive sampling or neural networks requires more powerful computational resources, while the direct matrix multiplication that we use is fairly easy to compute. Nevertheless, we expect that the joint use of these methods with our approach might be very interesting due to shorter data acquisition times and higher image resolution, and should be studied in the future. We also note that these estimates do not account for technical imperfections and delays, which depend on particular hardware equipment and software implementation, and reduce the final image resolution.

## 5. Results

### 5.1. Images of Variable Resolutions

**Figure 3:**
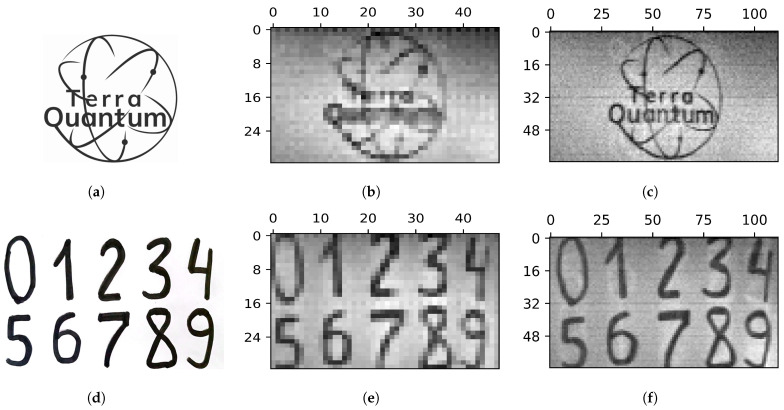
Objects (**a**,**d**), and their images of different resolutions. A 32×48 resolution is obtained in 24 steps via a sequence of 32×2 patterns (labeled by (**b**,**e**)). A 64×112 resolution is obtained in 112 steps via a sequence of 64×1 patterns (labeled by (**c**,**f**)).

A series of measurements of objects was carried out. Patterns made up of a 64×64 Hadamard matrix were used (the total number of patterns used was 128). In Figure 3, the results of visualization with two different resolutions are presented.

We obtain a 32×48 image resolution in 24 steps using a sequence of 32×2 patterns in discrete sweep mode. A resolution of 64×112 is obtained in 112 steps using a sequence of 64×1 patterns in continuous sweep mode. The number of 112 steps is selected based on the horizontal size of the object.

**Figure 4:**
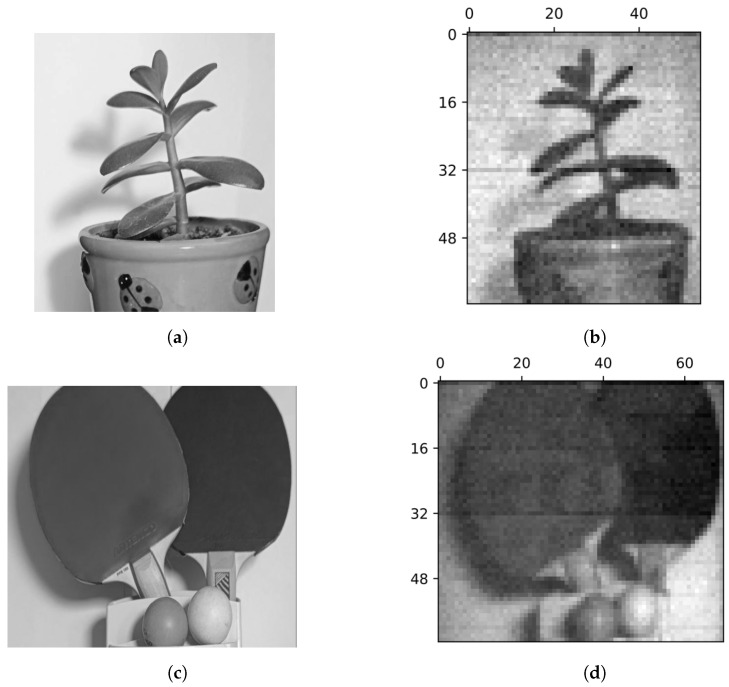
Examples of real-world images. A flower (**a**) and table tennis rackets (**c**) and their SPI images (**b**,**d**). The image of a flower (**b**) was obtained in 55 steps and has a resolution of 64×55. The image of table tennis rackets (**d**) was obtained in 70 steps and has a resolution of 64×70.

In Figure 4, we also present real-world images of more complicated objects to showcase the effectiveness of the proposed method. Note the smooth greytone transitions and natural shadows. However, we can notice some distortions in the presented images, for example, horizontal dark lines. In the next subsection, we investigate the origin of these distortions using simplified test objects. We also discuss possible solutions to mitigate this problem.

### 5.2. Image Artifacts

**Figure 5:**
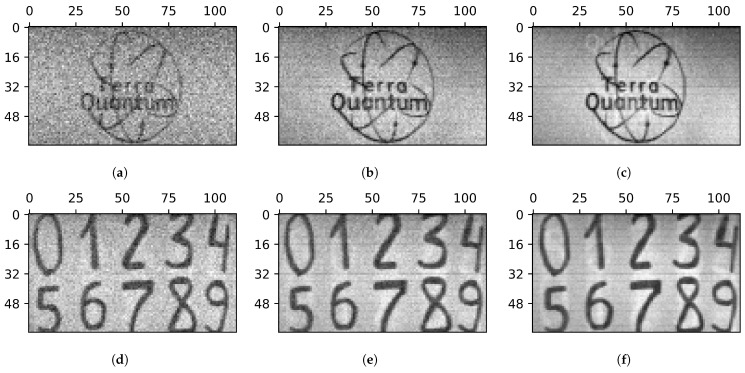
Images obtained under different illumination levels: natural ambient daylight (**a**,**d**); lamp power of 10.6 W (**b**,**e**); lamp power of 17.4 W (**c**,**f**). All images were obtained in 112 steps by a sequence of 64×1 patterns, and the image resolution is 64×112.

Within this subsection, we consider 64×112 images obtained using 128 patterns with a 64×1 resolution in continuous sweep mode. To clarify the required level of illumination, we investigate the dependence of the image quality on the illumination brightness. The illumination level was controlled by the voltage applied to the halogen lamp. Examples of the reconstructed images are shown in Figure 5. As the illumination level increases, the noise level becomes significantly lower, but even the natural ambient daylight is enough to obtain basic information about the black-and-white image.

**Figure 6:**
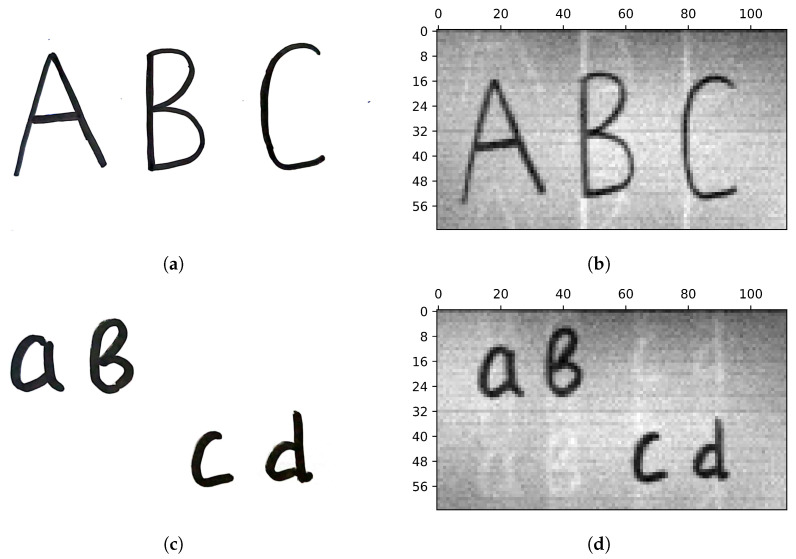
Comparison of objects (**a**,**c**) and their images (**b**,**d**). The image resolution is 64×112, and the power on the lamp used for illumination is 14.9 W. The images show artifacts expressed in random noise, horizontal dark lines, and “ghost” copies of letters. Horizontal dark lines are especially visible on vertical pixel numbers 0 and 32. “Ghosts” are located symmetrically relative to the middle of the image. For example, in (d), the secondary images of the letters “a” and “b” are below the original ones, and the copies of letters “c” and “d” are above them.

Figure 5 indicates the presence of image artifacts that become more visible at higher illumination brightness. Two types of artifacts are noticeable: light secondary images (copies) of dark areas of the object (in our case, the outlines of numbers and letters) and horizontal dark lines. These artifacts are more visible in the images of handwritten letters shown in Figure 6.

Since the patterns we use are one-dimensional, the horizontal dark stripes correspond to errors in fixed pixel positions that repeat on each measurement step and are independent of a specific object. Similar artifacts in fixed pixel positions were previously observed when reconstructing images using two-dimensional patterns based on Hadamard matrices in [23,24]. The presence of copies was noticed when using patterns in the order corresponding to the Sylvester construction of Hadamard matrices in [25].

To find out the origin of these artifacts, we measured the signal from the photodetector using an oscilloscope. The pattern sequence displayed on the DMD consists of three parts: the original patterns used for imaging, negatives of the original patterns (which are necessary due to the features of the DMD, as noted earlier), and the dark time interval. We add the dark time interval before the original pattern sequence to reduce the image distortion, as discussed below. During the dark time interval, no pattern is shown (all pixels are closed).

Since the original patterns have active and idle parts, and the idle part occupies most of the pattern area, the original patterns consist mostly of closed pixels. Therefore, the negatives consist mostly of open pixels (the negative of the idle part is filled by open pixels). This should lead to approximately the same level of light intensity within each of the two parts, with a sharp change between these parts. The measured signal during the dark time interval should be approximately of the same level as during the original pattern sequence. Thus, we should see two distinct levels of signal, with a sharp transition between them.

**Figure 7:**
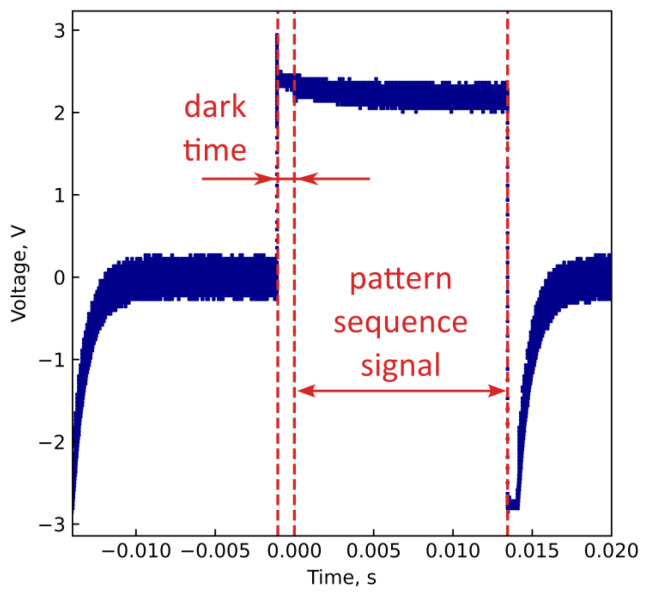
An example of an oscilloscope-measured signal from the photodetector corresponding to the entire sequence of patterns displayed on the DMD. The detector signal is inverted due to the features of the electrical circuit used to acquire the photodetector signal: a higher light intensity corresponds to a lower voltage. The part used for visualization is annotated. The dark time interval of 1 ms precedes the pattern sequence. Non-annotated parts of the signal correspond to the signal from the sequence of pattern negatives. The signal from the sequence of patterns used for imaging shows a slow decrease in the average voltage value, and the signal from the negatives shows a sharper increase.

In fact, we can see a slightly different signal waveform in Figure 7. Sharp intensity changes between each of two intensity levels are accompanied by a distortion of the waveform. The transition between two intensity levels does not occur instantaneously and is accompanied by smooth changes in the average value.

Possible reasons for the distortion of the waveform are related to the features of the photodetector and an electrical circuit for receiving and amplifying the signal. With a sharp change in the intensity of the incident light, the value of the photodetector signal does not change instantly, but requires a certain relaxation time. Thus, a sharp transition between different intensity levels can cause significant waveform distortions. This can also lead to correlations between successive measurements.

As we can see from the experiment, such distortions are observed for all objects. The waveform distortions do not make the image reconstruction impossible, although they can lead to imaging artifacts. This is due to the fact that the information about a particular object is contained in small signal changes compared to the average signal level. These changes are too small to be visible in Figure 7.

We reduce the image distortions by two methods. First, we adjust the pattern order in the displayed sequence: pairs of patterns corresponding to the values that are subtracted during reconstruction according to Equation (1) are located next to each other. The effect of slowly varying the signal component is significantly less between the adjacent measurements than between more distant ones. Subtraction partially removes this nearly constant signal component, thus reducing the effect of the slowly changing average on the reconstruction process. Second, we add the dark time interval before the original pattern sequence. The dark time allows for some signal stabilization before the measurements of the signal from the imaging patterns.

Possible methods of further artifact mitigation are related to hardware modifications. The photodetector in our setup has a fairly long relaxation time due to its large electrical capacity. In addition, high-capacity electronic components can also contribute. Thus, one possible solution is to use a detector with a lower capacitance and to improve the electrical circuit. Another solution is to use another spatial light modulator (instead of the DMD that we use) which would not require auxiliary patterns: the presence of auxiliary “negatives” of the original patterns is the primary source of the signal waveform distortions.

### 5.3. Computer Simulation

To verify whether the above-described waveform distortions actually cause the artifacts that are visible in the resulting images, we conduct a computer simulation. We use a photograph of handwritten letters as an object. The image resolution is set to 64×112.

**Figure 8:**
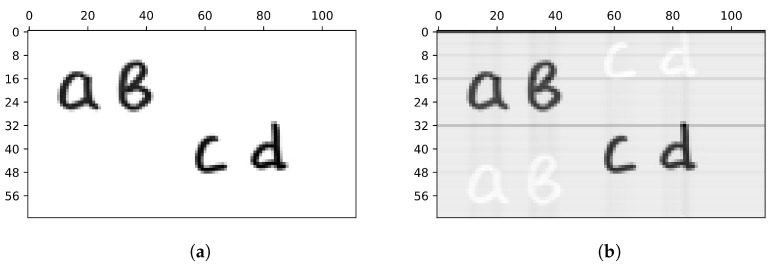
The object (**a**) and the result of a computer simulation to obtain its image (**b**). Correlations between the adjacent signal values and a slowly varying signal envelope are added to the ideal calculated signal. As a result of the simulation, the same image artifacts were obtained as in the real experiment, namely, dark horizontal lines and “ghost” copies of letters.

During the simulation, the values of signals $S^{+}$ and $S^{-}$ are calculated for each of the 112 columns of the object by multiplying the matrices of the patterns $H^{+}$ and $H^{-}$ by each column. We add correlations between successive measurements and a smooth change in the average. The exponent is chosen as a model of a smoothly varying average due to its visual similarity to the distortions observed in the Figure 7. An image of the object is obtained by multiplying the differences in the corresponding signal pairs by the inverse Hadamard matrix using Equation (1). The result of the simulation is shown in Figure 8. The simulation demonstrates similar distortions as we observe in the real experiment. The computer simulation also confirms that the waveform distortion leads to horizontal dark stripes, and the presence of “ghost” copies and background darkening can be explained by correlation between the adjacent measurements.

The values of the above-mentioned small signal changes that contain information about the object increase significantly with an increase in the illumination level, which leads to random noise reduction. At the same time, a higher illumination level causes sharper changes in the signal and, consequently, larger waveform distortions. Thus, increasing the illumination level reduces the image noise but leads to more visible image artifacts.

## 6. Conclusions

We have shown an experimental SPI method that allows us to obtain variable resolution while keeping the same number of light patterns. To demonstrate the proof of concept and study its practical application, we use patterns based on a 64×64 Hadamard matrix. We use a complete measurement and inverse matrix multiplication for image reconstruction. Within the standard SPI Hadamard approach, this allows us to obtain only 8×8 image resolution. Within our approach, we obtain images of 64×112 image resolution. We found imaging artifacts (which are also visible in previous works by other groups), and explained their origin for possible mitigation.

The presented approach is versatile and allows for various modifications of both software and hardware parts. On the algorithmic side, we plan to extend our approach to other image reconstruction methods beyond the Hadamard matrix multiplication. On the hardware side, the module with the rotating mirror can be modified to provide better timing or adjustable field of view.

## Data Availability

The data presented in this study are available on reasonable request from the authors.

## Abbreviations

The following abbreviations are used in this manuscript:

SPI	Single-pixel imaging
DMD	Digital micromirror device
ADC	Analog-to-digital converter
MCU	Microcontroller unit
PD	Photodetector

## References

[1] Sloane N.J.A., Harwit M. (1976). Masks for Hadamard transform optics, and weighing designs. Appl. Opt..

[2] Golay M.J.E. (1949). Multi-Slit Spectrometry. J. Opt. Soc. Am..

[3] Decker J.A., Harwitt M.O. (1968). Sequential Encoding with Multislit Spectrometers. Appl. Opt..

[4] Decker J.A. (1970). Hadamard–Transform Image Scanning. Appl. Opt..

[5] Decker J.A. (1971). Experimental Realization of the Multiplex Advantage with a Hadamard-Transform Spectrometer. Appl. Opt..

[6] Harwit M. (1971). Spectrometric Imager. Appl. Opt..

[7] Swift R.D., Wattson R.B., Decker J.A., Paganetti R., Harwit M. (1976). Hadamard transform imager and imaging spectrometer. Appl. Opt..

[8] Pittman T.B., Shih Y.H., Strekalov D.V., Sergienko A.V. (1995). Optical imaging by means of two-photon quantum entanglement. Phys. Rev. A.

[9] Candès E.J., Romberg J.K., Tao T. (2006). Stable signal recovery from incomplete and inaccurate measurements. Commun. Pure Appl. Math..

[10] Donoho D. (2006). Compressed sensing. IEEE Trans. Inform. Theory.

[11] Gibson G.M., Johnson S.D., Padgett M.J. (2020). Single-pixel imaging 12 years on: A review. Opt. Express.

[12] Wang Y., Huang K., Fang J., Yan M., Wu E., Zeng H. (2023). Mid-infrared single-pixel imaging at the single-photon level. Nat. Commun..

[13] Gibson G.M., Sun B., Edgar M.P., Phillips D.B., Hempler N., Maker G.T., Malcolm G.P., Padgett M.J. (2017). Real-time imaging of methane gas leaks using a single-pixel camera. Opt. Express.

[14] Stantchev R.I., Yu X., Blu T., Pickwell-MacPherson E. (2020). Real-time terahertz imaging with a single-pixel detector. Nat. Commun..

[15] Shcherbatenko M., Elezov M., Manova N., Sedykh K., Korneev A., Korneeva Y., Dryazgov M., Simonov N., Feimov A., Goltsman G. (2021). Single-pixel camera with a large-area microstrip superconducting single photon detector on a multimode fiber. Appl. Phys. Lett..

[16] Osorio Quero C.A., Durini D., Rangel-Magdaleno J., Martinez-Carranza J. (2021). Single-pixel imaging: An overview of different methods to be used for 3D space reconstruction in harsh environments. Rev. Sci. Instrum..

[17] Duarte M.F., Davenport M.A., Takhar D., Laska J.N., Sun T., Kelly K.F., Baraniuk R.G. (2008). Single-pixel imaging via compressive sampling. IEEE Signal Process. Mag..

[18] Zhao M., Li F., Huo F., Tian Z. (2022). Generative adversarial network-based single-pixel imaging. J. Soc. Inf. Disp..

[19] Zhou L., Bai Y., Fu Q., Zhu X., Huang X., Zou X., Fu X. (2024). Measurable speckle gradation Hadamard single-pixel imaging. Chin. Opt. Lett..

[20] Pratt W., Kane J., Andrews H. (1969). Hadamard transform image coding. Proc. IEEE.

[21] Hotelling H. (1944). Some improvements in weighing and other experimental techniques. Ann. Math. Stat..

[22] Hedayat A., Wallis W.D. (1978). Hadamard Matrices and Their Applications. Ann. Stat..

[23] Xiao Y., Zhou L., Chen W. (2019). Direct Single-Step Measurement of Hadamard Spectrum Using Single-Pixel Optical Detection. IEEE Photonics Technol. Lett..

[24] Sun R., Long J., Ding Y., Kuang J., Xi J. (2023). Hadamard Single-Pixel Imaging Based on Positive Patterns. Photonics.

[25] Vaz P.G., Amaral D., Ferreira L.F.R., Morgado M., Cardoso J. (2020). Image quality of compressive single-pixel imaging using different Hadamard orderings. Opt. Express.

